# Biofeedback efficacy for outlet dysfunction constipation: Clinical outcomes and predictors of response by a limited approach

**DOI:** 10.1111/nmo.14948

**Published:** 2024-10-25

**Authors:** Christian Lambiase, Massimo Bellini, William E. Whitehead, Stefan Lucian Popa, Riccardo Morganti, Giuseppe Chiarioni

**Affiliations:** ^1^ Gastrointestinal Unit, Department of Translational Research and New Technologies in Medicine and Surgery University of Pisa Pisa Italy; ^2^ NIHR Nottingham BRC Nottingham University Hospitals NHS Trust and the University of Nottingham Nottingham UK; ^3^ Nottingham Digestive Diseases Centre, Translational Medical Sciences, School of Medicine University of Nottingham Nottingham UK; ^4^ UNC Center for Functional GI and Motility Disorders University of North Carolina at Chapel Hill Chapel Hill North Carolina USA; ^5^ 2nd Medical Department Iuliu Hațieganu University of Medicine and Pharmacy Cluj‐Napoca Romania; ^6^ Clinical Trial Statistical Support Unit Azienda Ospedaliero Universitaria Pisana Pisa Italy; ^7^ Department of Medicine, Gastrointestinal Unit, Il Cerchio Med HealthCare Verona Center Verona Italy

**Keywords:** biofeedback, constipation, digital rectal examination, dyssynergic defecation, functional defecation disorders, irritable bowel syndrome, rectocele

## Abstract

**Background:**

Functional defecation disorders (FDD) are a common etiology of refractory chronic constipation (CC). FDD diagnosis (dyssynergic defecation [DD] and inadequate defecatory propulsion [IDP]), requires diagnostic tests including anorectal manometry (ARM) and balloon expulsion test (BET). Biofeedback (BF) is the treatment of choice for DD. The aims of our study were to evaluate: the outcome of BF in a group of constipated patients with defecatory disorders of any etiology; the efficacy of two simple diagnostic tools in predicting BF outcome in the short‐term.

**Methods:**

One hundred and thirty‐one refractory CC patients failing the BET underwent BF therapy. Before BF, all patients underwent the following:

- ARM.
- Straining questionnaire. The answers were: “belly muscles”; “anal muscles”; “both”; “Don't know/No answer.”
- Digital rectal examination augmented by abdominal palpation on straining (augmented‐DRE).

The BF therapist was blinded to ARM, straining questionnaire, and augmented‐DRE results.

**Key Results:**

Eighty‐one patients responded to BF. Gender, age, and IBS‐C showed no significant impact on BF response. Both DD and IDP responded equally to BF, while the rate of response in patients with isolated structural pelvic floor abnormalities was lower (*p* < 0.001). The answer “anal muscles” to straining questionnaire showed a strong association with BF response (*p* < 0.001). A lack in abdominal contraction and in anal relaxation on augmented‐DRE were strongly associated with BF response (*p* < 0.01). Absence of manual maneuvers to facilitate defecation was associated with BF response (*p* < 0.001).

**Conclusions & Inferences:**

BF is the therapy of choice for refractory constipation due to FDD of any etiology, inducing both clinical and anorectal physiology improvement in the short term. Comorbid IBS‐C did not affect outcome while symptomatic isolated pelvic floor abnormalities appeared refractory to behavior treatment.

The straining questionnaire and augmented‐DRE outcomes showed a strong correlation with BF response and can be implemented in clinical practice to improve the management of constipated patients by prompting early referral to BF.

## Graphical abstract

Biofeedback (BF) is the therapy of choice for functional defecation disorders of any etiology. BF was reported as an effective short‐term cure for constipation in up to 63% of patients. A straining questionnaire and a digital rectal examination augmented with abdominal palpation on straining were associated to BF response.

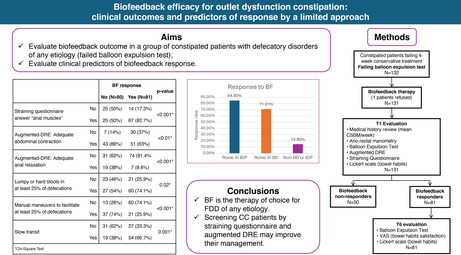

**Key points**

- Biofeedback is the therapy of choice for functional defecation disorders of any etiology, providing both clinical and physiology improvement.
- Biofeedback was reported as an effective short‐term cure for constipation in up to 63% of patients according to the remission of the Rome diagnostic III criteria.
- A straining questionnaire and a digital rectal examination augmented with abdominal palpation on straining were associated to BF response.

## 1 INTRODUCTION

Chronic constipation (CC) is defined by infrequent bowel movements and/or defective rectal expulsion symptoms.^1^ CC affects up to 11.7% of people worldwide^2^ and is one of the most common complaints for a referral to a gastroenterologist.^3^

In the absence of alarm symptoms, many patients require minimal diagnostic procedures and will respond to lifestyle modifications, diet advice, and the judicious use of laxatives. Additional colo‐rectal physiology tests are helpful to tailor treatment for refractory patients.^4^ However, these tests are often not available outside tertiary referral centers.^5^

Dyssynergic defecation (DD) is a functional defecation disorder (FDD) defined by the Rome III criteria as paradoxical contraction or inadequate relaxation of the pelvic floor muscles on straining.^1^, ^6^ The diagnosis of a Rome III FDD requires two concordant ano‐rectal physiology tests.^1^, ^6^ The diagnosis of subtypes of FDD, DD, and inadequate defecatory propulsion (IDP), usually requires ano‐rectal manometry (ARM) and a balloon expulsion test (BET).^1^, ^6^ Biofeedback (BF) is the treatment of choice for refractory constipation due to DD^4^ and has been reported to be effective,^7^, ^8^, ^9^ with a median response rate of approximately 67%.^7^ Randomized clinical trials have shown BF to be superior to sham‐BF, placebo pill, diazepam, and osmotic laxatives in patients with DD.^10^, ^11^, ^12^

Data available regarding BF's efficacy for other subtypes of evacuation disorders, such as IDP, or outlet dysfunction due to pelvic floor anatomical abnormalities are still lacking or conflicting.^13^ It is also unclear whether comorbid irritable bowel syndrome with predominant constipation (IBS‐C) may influence BF outcome for evacuation disorders.^14^

We recently provided evidence that two simple diagnostic tools were both effective in predicting a Rome III FDD diagnosis in refractory constipation^15^:

- A straining questionnaire;
- A digital rectal examination augmented by abdominal palpation in straining (augmented‐DRE).

We have provided evidence that ano‐rectal physiology tests and constipation subtype may be predictive of BF's outcomes,^10^ even in the absence of conclusive data.^10^, ^16^ However, no strong predictor of BF outcomes has been identified for all refractory constipation due to evacuation disorders.

The aims of our study were:

- To evaluate short‐term clinical and ano‐rectal physiology tests outcomes of BF in a group of refractory constipated patients due to evacuation disorders, as suggested by failed BET;
- To test the efficacy of augmented‐DRE, straining questionnaire and ano‐rectal physiology tests for predicting BF outcomes in the short‐term.

## 2 METHODS

### 2.1 Study population

Patients over 18 years‐old referred to a tertiary care center in Northern Italy for diagnosis and management of CC and/or IBS‐C, were screened between March 2010 and May 2012. All patients enrolled had a history of constipation lasting at least 1 year and met the Rome III criteria for functional constipation (FC) and/or IBS‐C.^1^, ^17^ Exclusion criteria were: history of abdominal surgery (excluding appendectomy or cholecystectomy), eating or psychological disorders, features of megarectum, megacolon, or chronic intestinal pseudo‐obstruction, endocrine disorders such as hypothyroidism or diabetes, regular use of drugs which may cause constipation.

All patients underwent a 4‐week trial of conservative treatment as part of the standard care: recommendations to increase fiber as tolerated up to 30 grams/day, fluids as tolerated up to 8 glasses/day, exercise as tolerated, and to take laxatives (including macrogol), enemas, or suppositories no more than twice/week. During the 4‐week trial of conservative treatment and after BF therapy, clinical information related to complete spontaneous bowel movements (CSBM), use of laxatives and symptoms through a bowel and symptom diary were collected from all patients. Laxatives were not allowed more than twice a week before BF therapy. After treatment, laxatives were allowed without limitation to better understand the effect of BF on symptoms.

The population included in the study is the same enrolled in previously published research.^15^ This group of patients had been evaluated from a diagnostic point of view as reported in a previous paper.^15^ In the current study, our aims were to assess the therapeutical response to BF and the predictive value of clinical features on BF outcomes.

### 2.2 Study design

This is a nonrandomized open study. Patients were enrolled after the signing of informed consent. The study was conducted according to the ethical principles of the Declaration of Helsinki and to good clinical practice (GCP). Final approval was granted by the Ethics Committee of the Azienda Ospedaliera Universitaria Integrata di Verona Committee (CE 2158).

#### 2.2.1 Medical examinations and BF

Patients who failed a four‐week conservative treatment trial were evaluated at first medical examination (T0).

At T0, the patients were asked to fill in a questionnaire including the Rome III criteria for FC/IBS‐C^1^, ^17^ and a self‐administrated straining questionnaire. At the same visit were performed:

- Augmented‐DRE;
- BET;
- Conventional low‐resolution water‐perfused ARM;
- Colonic transit time (CTT) with radiopaque markers.

Perception of patient's satisfaction of their bowel habits was assessed through a 100 mm linear visual analogue scale (VAS) ranging from 0 to 100 (0 = completely satisfied; 100 = completely unsatisfied). The mean use of laxatives/week and the mean CSBM/week were registered before and after BF therapy.

Patients whose BET and ARM were inconsistent underwent a barium defecography, performed according to the Faccioli et al. technique.^18^ The following findings were considered consistent with structural outlet obstruction: (1) rectocele greater than 4 cm (grade III), (2) intussusception extending into the distal anal canal, (3) prolapse of rectal mucosa beyond the anus, (4) enterocele that appeared to obstruct rectal emptying with retained contrast at the end of straining.

Using these data, patients were assigned to five diagnostic subtypes (Table 1).

**TABLE 1:** Comparison between demographic features and constipation groups. No differences were observed regarding sex among the groups. Patients with DD had a lower mean age compared with all the other groups (*p* < 0.001). Patients with an isolated slow transit constipation had a lower median age than patients with isolated structural outlet obstruction (*p* < 0.01). Statistics: Frequency (%) or mean (±SD).

Demographic feature	DD group (*N*. 69)	IDP group (*N*. 33)	Isolated structural outlet obstruction group (*N*. 27)	Isolated slow transit constipation group (*N*. 33)	Normal transit constipation group (*N*. 67)	*p*‐Value
Gender						
Male	7 (10.14%)	0 (0%)	2 (7.4%)	1 (3.03%)	8 (11.93%)	0.39^a^
Female	62 (89.86%)	33 (100%)	25 (92.6%)	32 (96.97%)	58 (86.57%)	
Age	39.67 (13.33)	45.6 (13.58)	53.44 (12.37)	43.48 (15.45)	49.28 (16.12)	<0.001^b^

Abbreviations: DD, dyssynergic defecation; IDP, inadequate defecatory propulsion; SD, standard deviation.

^a^ Chi‐Square Test.

^b^ ANOVA with Bonferroni correction.

Patients who failed the BET, regardless of colo‐rectal physiology tests, underwent BF therapy. Failure to evacuate a rectal balloon has been shown to correlate with both BF and improved posture outcome in refractory constipation due to defecatory disorders.^10^, ^19^

The BF treatment protocol, performed according to previously published techniques,^10^, ^11^ consisted of 5 weekly BF training sessions that lasted 30–45 min. Briefly, patients were.

First taught to strain more effectively and to coordinate expulsion efforts with their breathing. Next, they were taught to relax pelvic floor muscles during straining by electromyography (EMG) feedback imaging. A surface intra‐anal EMG probe connected to a portable instrument (Myotron‐120; Enting Instruments & Systems, Dorst, The Netherlands) was used to measure pelvic floor EMG responses to attempted defecation. A sustained increase in average EMG activity during straining trials greater than 50% above resting EMG levels (i.e., paradoxical contraction) was indicative of PFD. In the final phase of training, patients practiced defecating a 50‐mL, air‐filled balloon while the trainer gently pulled on the catheter connected to the balloon. After BF training, all patients were told that their pushing efforts had improved; this was done to ensure that patients entering the follow‐up phase of the study, had a positive expectation and would be motivated to return for follow‐up. All the BF training sessions were performed by a registered nurse unaware of physiology results, but BET.

One month after the end of BF therapy, patients were re‐assessed (T1). Patients were asked to fill out the straining questionnaire, the Rome III symptoms questionnaire for FC and VAS again and underwent for a second time augmented‐DRE, ARM, and BET.

We classified as BF responders only those patients meeting both of the two primary aims:

- An improvement of at least 1 CSBM/week compared to basal value (registered during the 4‐week conservative treatment). CSBM is considered an objective measure of response to therapy.^20^
- A score of 6 or 7 on a Likert scale expressing the defecation quality ranging from 1 to 7 (where 1 stands for “Markedly Worse”, 4 for “No Change” and 7 for “Markedly Better”). Likert scale is considered a reliable subjective measure of response to therapy.

Responders were re‐assessed after 6 months from the end of treatment (T6) with BET, VAS, and Likert.

#### 2.2.2 Straining questionnaire

Patient's perception of which muscles they predominantly used to push to defecate was evaluated using a self‐administrated standardized straining questionnaire: “What muscles do you mainly use when you push to defecate?” There were four possible answers: “abdominal muscles,” “anal muscles,” “both muscles,” “I don't know/no answer.” The questionnaire was created based on one of the authors' prior experiences (GC). The straining questionnaire showed very good reproducibility and the answer “anal muscles” was strongly associated with a FDD diagnosis.^15^

#### 2.2.3 Augmented‐DRE

For each patient, three push attempts were made, and the diagnosis was made based on two out of three of them. During each push attempt, both the anal relaxation and the abdominal contraction were recorded. Augmented‐DRE was performed by different gastroenterologists at different skill levels to replicate a real‐world situation.

Augmented‐DRE showed very good and moderate reproducibility regarding the evaluation of abdominal contraction and anal relaxation, respectively.^15^ Furthermore, both failed abdominal contraction and failed anal relaxation showed a strong association with a FDD.

#### 2.2.4 Anorectal manometry

Low‐resolution ARM was performed according to a previously published technique with patients in left lateral position.^15^, ^21^ The most relevant ARM variables were the rectal pressure and the anal pressure on straining.^1^ Rectal pressure was considered abnormal and supportive of IDP according to the Rome III criteria, if increased less than 45 mmHg on straining, while a Rome III DD diagnosis was considered when a paradoxical increment or less than >20% decrement from baseline in anal canal pressures was observed on straining.^1^

#### 2.2.5 BET and CTT

The BET was carried out before and after BF using a 16 Fr Foley catheter covered with surgical lubricant and filled with 50 mL of water at approximately 37°C. A Foley catheter was used because it is a standardized healthy device available in most medical clinic settings.^21^, ^22^, ^23^ The subjects were asked to evacuate the balloon within 2 min sitting on a commode in privacy.^21^, ^22^ BET was considered abnormal if patients were not able to expel it after 2 min.^21^, ^22^

CTT with radio‐opaque markers was performed with a single X‐Ray at 120 h while on a high‐fiber diet and refraining from laxatives, according to a previously published technique.^10^

### 2.3 Statistical analysis

Categorical data were described with absolute and relative (%) frequency. Continuous data were summarized with mean and standard deviation.

Categorical and continuous data were analyzed using the chi‐square test and *t*‐test for independent samples, Mann–Whitney test or one‐way ANOVA, followed by multiple comparisons with the Bonferroni method, respectively.

The Friedman test, the McNemar test and the Wilcoxon test were performed to assess repeated measures, when appropriate.

Multivariate analyses were based on the binary logistic regression.

Significance was set at 0.05 and all analyzes were carried out by SPSS v.29 technology.

## 3 RESULTS

### 3.1 Patients

Two hundred twenty‐nine patients failed the conservative treatment and were evaluated at T0 (Figure S1). All patients were Caucasian of Italian Heritage.

According to ARM, BET, CTT, and X‐Ray defecography, the Rome III criteria and pathophysiological mechanisms, patients were classified in five groups (Table 1). Twelve clinically significant rectoceles were identified (4 associated with an enterocele, 1 associated with a descending perineum, 2 associated with a descending perineum and enterocele).

CTT was delayed in 105 patients (29 patients with markers in the recto‐sigmoid colon (RS) and 76 with markers distributed in all the segments of the colon).^6^

One hundred thirty‐two patients failed the BET and 131 patients agreed to undergo BF. Out of these 131 patients, 85 showed 0 CSBM/week, 40 showed 1 CSBM/week and 6 showed 2 CSBM/week at baseline.

### 3.2 Biofeedback therapy

One hundred thirty‐one patients completed the BF treatment and all 131 were re‐evaluated at T1. Eighty‐four patients improved in the objective aim (CSBM), showing an increase of at least 1 CSBM compared to basal value. Details on the number of CSBM before and after BF are summarized in Table 2. Eighty‐one patients reported both subjective and objective improvement and were considered responders to BF.

**TABLE 2:** Mean number of CSBM before and after BF. Before BF, the majority of patients reported 0 or 1 mean CSBM per week, while after BF most patients reported 1 or 2 CSBP per week, with up to 16 of them reporting 3 or more CSBM/week. Statistics: Frequency (%).

	Before BF (*n* = 131)	After BF (*n* = 131)
0 CSBM/week	85 (64.89%)	19 (14.5%)
1 CSBM/week	40 (30.53%)	51 (38.93%)
2 CSBM/week	6 (4.58%)	45 (34.35%)
3 CSBM/week	—	15 (11.45%)
4 CSBM/week	—	1 (0.77%)

Abbreviations: BF, biofeedback; CSBM, complete spontaneous bowel movements.

Forty‐eight were nonresponders and 3 patients reported discordant results as regards the primary aim outcomes (improvement in the objective, but not subjective aims). Thirty‐one treated patients met the criteria for IBS‐C according to the Rome III criteria.^17^

#### 3.2.1 Biofeedback outcomes

On univariate and multivariate analysis, neither gender nor age had an impact on BF response. IBS‐C also showed no impact on the BF outcomes. The nonresponders group showed a higher prevalence of structural outlet dysfunction compared with responders (23 vs. 4, respectively, *p* < 0.001).

To explore BF outcomes in subtypes of outlet dysfunction, patients were classified according to the Rome III criteria. Both DD and IDP groups showed a high response rate to BF after 1 month (Table 3).

**TABLE 3:** Response to BF according to Rome III Criteria and etiology subgroups. Functional defecation disorders of all etiologies responded to BF therapy, whereas isolated structural outlet obstruction (e.g., rectocele >4 cm, intussusception extending into the distal anal canal, prolapse beyond the anus, obstructive enterocele with retained contrast at the end of straining) did not. Statistics: Frequency (%).

	Response to BF after 1 month		*p*‐Value^a^
	No (*N* = 50)	Yes (*N* = 81)	
Subgroups according to Rome III Criteria			
IDP (*N* = 33)	5 (15.2%)	28 (84.8%)	<0.001
DD (*N* = 69)	20 (28.9%)	49 (71.01%)	
Structural outlet obstruction alone (*N* = 27)	23 (85.2%)	4 (14.8%)	
Subgroups according to etiology			
DD + IDP (*N* = 22)	3 (13.6%)	19 (86.4%)	<0.001
DD alone (*N* = 69)	20 (28.9%)	49 (71.01%)	
IDP alone (*N* = 11)	2 (18.2%)	9 (81.8%)	
Structural outlet obstruction alone (*N* = 27)	23 (85.2%)	4 (14.8%)	

Abbreviations: BF, biofeedback; DD, dyssynergic defecation; IDP, inadequate defecatory propulsion.

^a^ Chi‐Square Test.

Patients were also classified according to predominant etiology underlying outlet dysfunction and BF response was re‐evaluated among these groups. No difference was observed among functional groups: patients with “DD alone” responded in a similar way to BF compared with patients with “IDP alone” or combined “DD + IDP.” In both cases, patients with isolated structural outlet obstruction had a rate of response to BF much lower compared with other groups (Table 3).

The 81 responders were all re‐assessed at 6 months after BF course (T6). On Likert scale at T6, 79 patients maintained a satisfactory bowel habit and 13 of the further improved compared to T1 Likert score. Only 2 patients reported a worsening from T1.

#### 3.2.2 Clinical outcomes of BF

**FIGURE 1:**
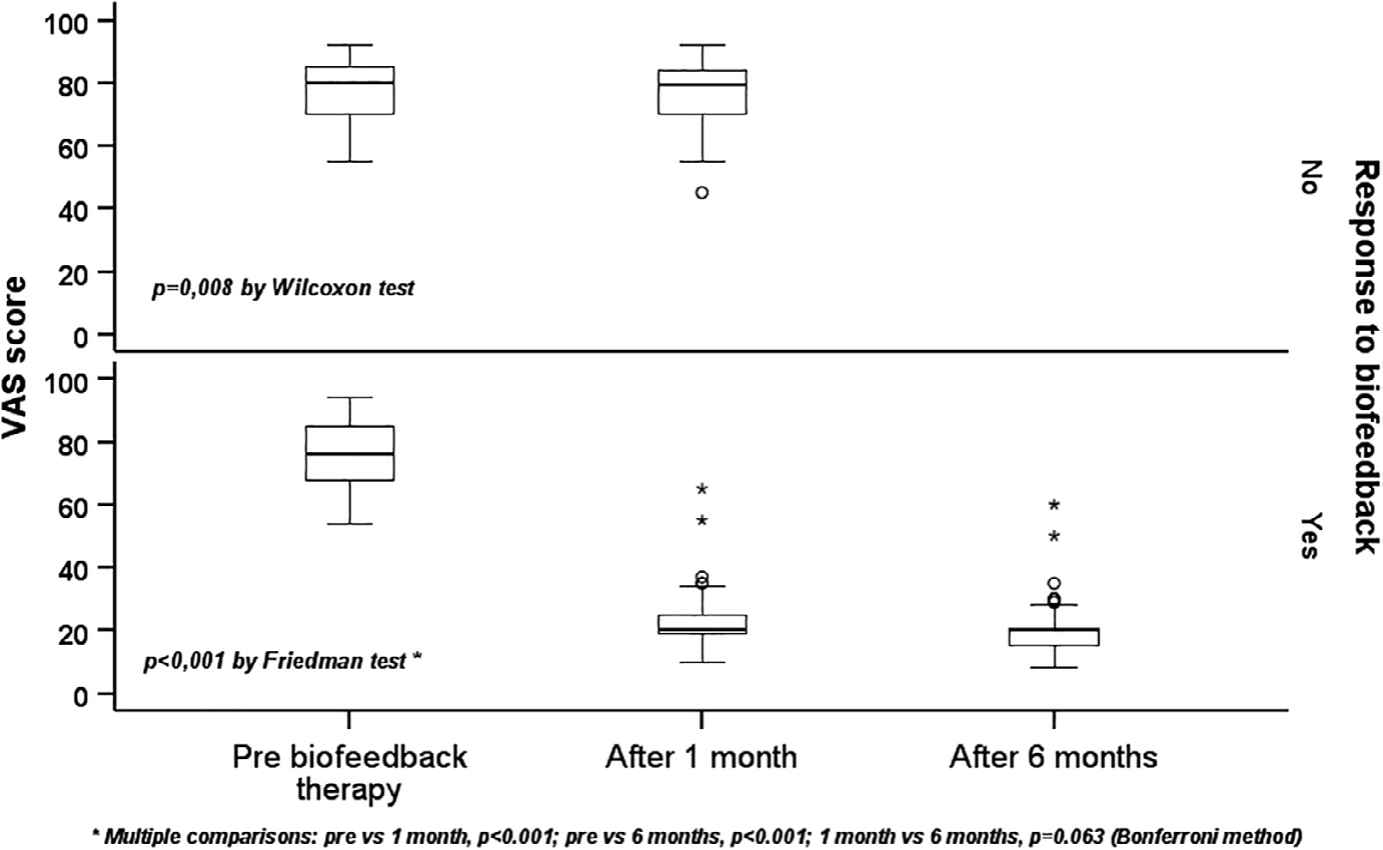
Quality of defecation assessed through VAS score. All 131 patients responded to VAS before and 1 month after BF. At 6 months, were re‐evaluated only responder patients (81). Responders to BF showed a significant improvement comparing the score before and 1 month after BF. Non responders showed a slight, although significant, improvement after BF. Friedman's and Wilcoxon's tests were used for comparisons.

There were no differences in median VAS scores between responders and nonresponders before BF (*p* = 0.8). An improvement in bowel satisfaction was registered through VAS after BF therapy in the responder group 1 month after the BF course (*p* < 0.001, Figure 1). Nonresponders showed a small, although significant, improvement in VAS (*p* = 0.008, Figure 1). Responders reported a slight, but not statistically significant, further improvement at 6 months (T6) (*p* = 0.063, Figure 1).

**FIGURE 2:**
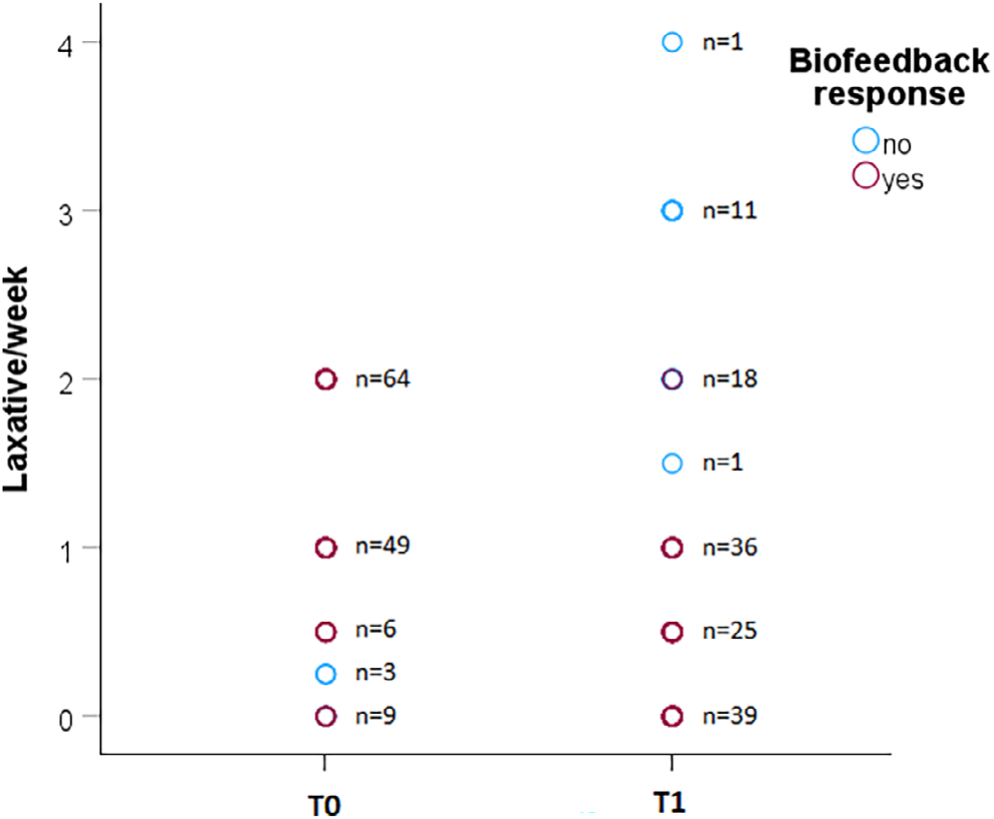
Scatter plot showing the use of laxatives before and after BF therapy in responders and nonresponders groups (81 vs. 50, respectively). Before BF therapy, laxatives were limited up to a maximum of two times per week, while after BF there were no limitations on the use of laxatives, to better assess BF response. Responders decreased the mean use of laxatives/week, while nonresponders increased the mean use of laxatives/week.

A significant decrease in the use of laxatives/week after BF was reported. Responders showed a marked decrease in number of laxatives/week, whereas nonresponders increased the number of laxatives/week (*p* < 0.001, Figure 2).

All Rome III FC symptoms showed a significant decrease in frequency 1 month after the end of BF therapy (Table S1). The symptom which showed the less improvement with BF was manual maneuvers to facilitate defecation.

At T1, 62.6% patients no longer met the Rome III criteria for a diagnosis of FC, showing less than two Rome III criteria among the six needed for a diagnosis of FC.

#### 3.2.3 Manometric outcomes of BF

At baseline, anal pressure during push was lower in nonresponders group compared to responders group. There were no other differences in manometric parameters and rectal sensory testing (RST) between responders and nonresponders. In the responder group, some manometric and all RST parameters showed a significant improvement after BF (Table 4). Regarding RST, those who responded to BF showed a greater decrease in volume for all three RST parameters. After BF, defecation threshold, rectal and anal pressures during push were significantly different between responders and nonresponders (*p* < 0.01). Nonresponders showed a significant improvement after BF only in the first sensation, defecation threshold and rectal pressure during push (*p* < 0.01).

**TABLE 4:** Manometric and RST parameters pre and post BF in responder (*N* = 81) and nonresponder (*N* = 50) groups. For all RST parameters and the push rectal pressure, there was a significant improvement after BF therapy in responders' group. Statistics: Mean (SD).

Manometric parameters	Response to BF	Before BF	After BF	*p*‐Value^b^
First sensation (mL)	Responders	24.57 (9.62)	19.14 (6.56)	<0.01
	NonResponders	24 (9.48)	21.8 (8.25)	0.017
	*p*‐Value^a^	0.725	0.063	
Defecation threshold (mL)	Responders	78.75 (31.6)	53.47 (13.43)	<0.01
	NonResponders	82.29 (37.88)	66 (23.56)	<0.01
	*p*‐Value^a^	0.781	<0.01	
Maximum tolerated volume (mL)	Responders	238.89 (81)	211.72 (38.95)	<0.01
	NonResponders	236 (86.33)	232 (57.82)	0.628
	*p*‐Value^a^	0.911	0.076	
RAIR elicited (mL)	Responders	17.77 (6.32)	17.03 (5.1)	0.095
	NonResponders	17.2 (7.01)	16.4 (5.63)	0.072
	*p*‐Value^a^	0.5	0.453	
Push rectal pressure (mmHg)	Responders	64.62 (31.41)	94.35 (13.65)	<0.01
	NonResponders	69.94 (22.47)	78.28 (20.79)	<0.01
	*p*‐Value^a^	0.228	<0.01	
Push anal pressure (mmHg)	Responders	66.11 (24.48)	37.25 (9.48)	<0.01
	NonResponders	51.84 (23.12)	46.04 (19.32)	0.21
	*p*‐Value^a^	<0.01	0.03	
Anal resting pressure (mmHg)	Responders	62.35 (11.6)	61.01 (11.9)	0.023
	NonResponders	58.96 (10.88)	58.32 (10.04)	0.144
	*p*‐Value^a^	0.135	0.158	

Abbreviations: BF, biofeedback; RAIR, recto‐anal inhibitory reflex; RST, rectal sensory testing; SD, standard deviation.

^a^ Mann‐Whitney Test between groups at T0 and at T1.

^b^ Wilcoxon Test between T0 and T1 in responders and nonresponders groups.

After BF:

- 55 Rome III DD pattern improved (79.71%), with a reverse to normal of dyssynergia on ARM.
- 31 Rome III IDP pattern improved (93.94%), with a normalization of rectal pressure on straining.
- 82 had a negative BET (62.6%).

At T6, among 81 responder patients, 80 showed a negative BET. All responders who were able to expel the balloon at T1 were also able to expel the balloon at T6.

Patients who improved in ARM or BET had a significantly higher response rate to BF compared to patients who did not improve (*p* < 0.001) (Table S2).

### 3.3 Predictors of BF response

The answer “anal muscles” to the straining questionnaire showed a strong association with BF response (Table 5, *p* < 0.001). Patients reporting anal straining had a higher rate of improvement if compared with patients who did not report anal straining (72.83% vs. 35.9%, respectively; *p* < 0.001).

**TABLE 5:** Association of straining questionnaire response, augmented‐DRE and Rome III questionnaire features associated to BF outcomes. Statistics: Frequency (%).

	BF response		*p*‐Value^a^
	No (*N* = 50)	Yes (*N* = 81)	
Straining questionnaire answer “anal muscles”			
No	25 (50%)	14 (17.3%)	<0.001
Yes	25 (50%)	67 (82.7%)	
Augmented‐DRE: Adequate abdominal contraction			
No	7 (14%)	30 (37%)	<0.01
Yes	43 (86%)	51 (63%)	
Augmented‐DRE: Adequate anal relaxation			
No	31 (62%)	74 (91.4%)	<0.001
Yes	19 (38%)	7 (8.6%)	
Lumpy or hard stools in at least 25% of defecations			
No	23 (46%)	21 (25.9%)	0.02
Yes	27 (54%)	60 (74.1%)	
Manual maneuvers to facilitate at least 25% of defecations			
No	13 (26%)	60 (74.1%)	<0.001
Yes	37 (74%)	21 (25.9%)	
Slow transit			
No	31 (62%)	27 (33.3%)	0.001
Yes	19 (38%)	54 (66.7%)	

Abbreviations: Augmented‐DRE, Digital rectal examination augmented by abdominal palpation on straining; BF, biofeedback.

^a^ Chi‐Square Test.

An inadequate abdominal contraction and an inadequate anal relaxation on augmented‐DRE were strongly associated with BF response (Table 5, *p* < 0.001). Patients showing an inadequate abdominal contraction significantly improved compared to patients who showed an adequate contraction (81.08% vs. 52.25%, respectively; *p* < 0.01). Patients showing an inadequate anal relaxation during straining significantly improved with BF compared to patients who showed an adequate anal relaxation (70.48% vs. 26.92%, respectively; *p* < 0.001). Regarding the Rome III FC symptom questionnaire, the only two symptoms which seem to predict a BF response with univariate analysis were lumpy or hard stools in at least 25% of defecations and the use of manual maneuvers to facilitate defecation in at least 25% of defecations (*p* = 0.02 and *p* < 0.001, respectively, Table 5). Patients with lumpy or hard stools and patients not using manual maneuvers to defecate were more likely to respond to BF.

We performed a multivariate analysis including all the identified factors associated with BF response: the only two independent factors associated with BF response were an inadequate abdominal contraction on augmented‐DRE and the nonuse of manual maneuvers to facilitate defecation (Table 6). These parameters may be considered to be predictors of BF response.

**TABLE 6:** Multivariate analysis of factors associated to BF response: The only features that made an independent contribution to outcome were digital facilitation of defecation, which predicted failure to BF, and inadequate abdominal contraction during push, which predicted success to BF.

	RC	OR (95% CI)	*p*‐Value
Straining questionnaire answer “anal muscles”: (0) No, (1) Yes	0.55	1.73 (0.55–5.43)	0.35
Augmented‐DRE: Adequate abdominal contraction: (0) No, (1) Yes	−1.52	0.22 (0.07–0.66)	0.007
Augmented‐DRE: Adequate anal relaxation: (0) No, (1) Yes	−0.8	0.45 (0.11–1.77)	0.25
Lumpy or hard stools in at least 25% of defecations: (0) No, (1) Yes	0.57	1.76 (0.61–5.13)	0.3
Manual maneuvers to facilitate at least 25% of defecations: (0) No, (1) Yes	−1.93	0.15 (0.06–0.36)	<0.001
Slow transit: (0) No, (1) Yes	0.54	1.71 (0.6–4.88)	0.13
*Constant*	1.69	5.41	0.02

Abbreviations: 95% CI, 95% confidence interval; Augmented‐DRE, Digital rectal examination augmented by abdominal palpation on straining; BF, biofeedback; OR, odds ratio; RC, regression constant.

Patients who reported the use of manual maneuvers had a lower response rate to BF compared to patients who did not (36.2% vs. 82.19%, respectively; *p* < 0.001). This criterion is associated with a poor outcome of BF therapy (*p* < 0.001).

Patients with slow transit had a significant improvement compared to patients with normal transit (73.92% vs. 46.55%, respectively). RS patients mostly improved after the BF course (82.14%) in comparison with other patients. These data are in line with previous studies.^10^

## 4 DISCUSSION

CC is a complex syndrome affecting a certain proportion of the population.^2^ In a subset of refractory CC patients, the diagnosis of Rome III DD is reached after a complex diagnostic approach requiring multiple tests.^4^ However, ano‐rectal physiology tests may not be available outside referral centers with CC patients repeatedly undergoing unnecessary procedures and laxative trials.^5^ In these subjects, BF is the most effective treatment.^4^ Few centers provide BF treatment and proper selection is the key to successful referral.

Having simple tools useful to predict a BF response may ease the workout of these patients with an access to adequate therapies, avoiding troublesome and expensive investigations or frustrating treatments for both patients and physicians.

In our study, constipated patients showing outlet dysfunction, documented by a failed BET, regardless of the etiology of outlet dysfunction, underwent BF therapy. To strengthen the evidence obtained in our study, responders were considered to be only those patients who met both primary aims: an improvement of at least 1 CSBM/week compared to basal value and an improvement of the defecation quality expressed through a score of 6 or 7 on a Likert scale.

We confirmed the efficacy of BF in the treatment of DD, as reported by previous studies.^10^, ^11^, ^12^, ^24^ Furthermore, our results have shown that BF is also effective on Rome III IDP. IDP, as identified by decreased/absent rectal pressure on straining, is acknowledged as relevant etiology of constipation, though poorly addressed in the literature.^4^, ^5^ Noteworthy, a controlled study employing high‐resolution manometry showed that DD, IDP, and a hybrid of both abnormalities were uncorrelated, suggesting that the pathophysiology of DD and IDP are potentially distinct.^25^ Moreover, a recent study reported on finding IDP in 57% of patients undergoing sacral neuromodulation for refractory constipation after pelvic surgery.^26^ To the best of our knowledge, this is the first trial demonstrating the efficacy of BF for all the subtypes of FDD. In addition, our data may support an update of the Rome Criteria when confirmed by additional studies.

BF is effective in the short term (up to 6 months) for all forms of Rome III FDD, without rehearsing sessions.

IBS‐C showed no impact on BF response. These data are in line with a recent study demonstrating the efficacy of BF in improving clinical condition and quality of life in patients with pelvic floor dyssynergy with IBS.^27^ Therefore, we strongly advise considering coexisting FDD in patients consulting for IBS‐C to improve their management by means of BF therapy.

BF was reported as an effective short‐term cure for constipation in up to 63% of patients: these patients no longer met the Rome diagnostic III criteria after treatment (Table S3). The decreasing use of laxatives/week after therapy was again in line with the results reported above. The increased use in nonresponders may be due to frustration associated with an ineffective therapy.

Regarding the VAS score, we observed a significant decrease in median score after BF therapy in both responders (76 [17] before and 20 [6] after) and nonresponders groups (80 [14.3] before and 79.5 [13.8] after, Figure 1). This statistical data is discordant with our primary aims. It is well known that a statistical improvement does not always correspond to clinically significant improvement and that in DGBIs the contact with the clinician can give a positive perception on symptoms. We believe this slight improvement may be related to a placebo effect: all patients were told after BF that their pushing effort had improved in order to facilitate adherence to follow‐up.

We observed a reduction in the RST parameters. In this regard, no specific sensory retraining was provided to patients. The observed improvement in RST may be related to an improvement in global rectal function. These changes in sensory thresholds are believed to reflect the effects of eliminating or reducing the chronic distention of the rectum with retained stool.^10^ Although this was beyond the aims of our study, these results confirm our previous experience with BF.^11^ Whether the observed ARM and RST improvement is a direct consequence of BF therapy or of a global bowel function improvement could not be defined by our study.

We have demonstrated that it is possible to select constipated patients who have more chance of improving with BF therapy. The straining questionnaire, the augmented‐DRE and some clinical features (digitation to facilitate defecation and hard stools) were associated with BF response. However, these features were correlated; the only features that made an independent contribution to outcome were digital facilitation of defecation and adequate abdominal contraction during push, which predicted failure to BF. The use of manual maneuvers may be utilized as a clinical predictor of a scarce response to BF and physicians should evaluate an alternative therapy for these patients. These data confirm the findings from our previous randomized trial.^11^

CTT, when available, may be a useful tool to further screen refractory CC: evidence of slow transit (especially RS) is strongly associated with BF response. Several studies have indeed linked this specific CTT pattern to evacuation disorders.^28^, ^29^ These data are also in line with our former report on BF efficacy for slow transit constipation.^10^ Although slow transit was associated with BF response, our data do not support that CTT may be used as a predictor of BF response.

Predictive values of our proposed tools are at variance with other recently published studies where anal sphincter pressures predicted BF outcomes in community practice constipated patients.^16^ The discrepancy may be explained by a different patient selection as we chose to study tertiary care referral subjects.^16^ Moreover, high resolution anorectal manometry was not available in our study.

Rectoceles and allied disorders may be a relevant etiology of refractory constipation.^13^ The best management for these disabled patients is still a matter of debate. The failure of retraining for this hard‐to‐treat group of patients provides additional support to the multidisciplinary approach recently suggested for improving management.^13^ Moreover, a failed BET may strengthen surgical referral in the presence of both refractory constipation and isolated morphological abnormalities of the pelvic floor.^13^

The present study has some limitations.

Firstly, this was an open‐label clinical study without a control group and we cannot confidently exclude a placebo effect. Moreover, all patients after BF were told that their pushing effort had improved in order to facilitate compliance with follow‐up. We acknowledge that this may have had a positive impact on patients' subjective evaluation of treatment outcome (Figure 1).

Secondly, barium defecography was performed only in patients who had discordant results of BET and ARM, as suggested by the Rome III criteria, due to ethical concerns.

Thirdly, this study was conducted approximately 10 years ago, using a low‐resolution perfused catheter to perform ARM and using the Rome III criteria: using the Rome IV Criteria and a high‐resolution/high‐definition ARM would be very interesting in order to confirm (or not) our results.

Furthermore, the study included predominantly females. This may be due to the higher prevalence of the disorder in this gender.^2^ Although gender was not a significant factor in multivariate analysis, the data needs to be confirmed by larger studies.

Moreover, BET was performed using a 16 Fr Foley catheter, a technique previously validated in other studies.^21^, ^22^ However, other defecatory devices could lead to a different selection of patients given that the Foley catheter BET has been reported as having failed in a small percentage of healthy Australian volunteers.^30^ Lastly, augmented‐DRE was performed by different practitioners with different levels of training making our results sensitive to examiner' bias. However, augmented‐DRE may be potentially more effective on screening FDD in constipation when performed by skilled Personnel.

BF is the therapy of choice for Rome III FDD of any etiology, providing both clinical and physiology improvement. We also confirmed that comorbid IBS‐C did not influence treatment outcomes. To the best of our knowledge, this is the first trial investigating the efficacy of BF for refractory CC due to all subtypes of Rome III FDD, suggesting an early referral regardless the subtype of FDD. BET may both guide treatment and be used as a marker of successful BF, but additional ano‐rectal physiology tests provide meaningful diagnostic refining, especially in nonresponders to BF or in patients reporting the use of manual maneuvers.

## 5 CONCLUSIONS

Functional defecation disorders identification is key to effective patient management. Based on our results, we propose two simple tools to predict BF response in patients with failed BET: the straining questionnaire and the augmented‐DRE. The evaluation of manual maneuvers may add significant information regarding the likelihood of the success of BF.

Our data suggest that refractory constipation may be evaluated by augmented‐DRE to improve their management by prompting referral to BF in centers with limited access to ano‐rectal physiology testing, as it resulted to be a predictor of BF response. The evaluation of digital maneuvers to facilitate defecation may better define who may respond to BF. Moreover, the straining questionnaire is a less effective, alternative option as a predictor of BF response for those who are embarrassed by the procedure.

## AUTHOR CONTRIBUTIONS

G.C. had a role in conceptualization, data curation, methodology, supervision, investigation, writing the draft manuscript, and approved the final version. C.L. had a role in conceptualization, data curation, formal analysis, writing the draft manuscript, and approved the final version. W.E.W had a role in conceptualization, formal analysis, methodology, supervision, writing the draft manuscript, and approved the final version. R.M. had a role in formal analysis, methodology, and approved the final version. S.L.P had a role in conceptualization, drafting the manuscript, and approved the final version. M.B. had a role in conceptualization, formal analysis, supervision, writing the draft manuscript, and approved the final version. All authors have approved the final version of the manuscript.

## FUNDING INFORMATION

No funding declared.

## CONFLICT OF INTEREST STATEMENT

All authors have no conflicts of interest to be declared.

## GUARANTOR OF THE ARTICLE

Giuseppe Chiarioni, MD, Prof.

## Supporting information


Appendix S1.

## Data Availability

The data that support the findings of this study are available from the corresponding author upon reasonable request.
