# Supplementary material for: Biofeedback efficacy for outlet dysfunction constipation: Clinical outcomes and predictors of response by a limited approach
Source: Neurogastroenterol Motil. 2024 Oct 25;37(1):e14948. doi: 10.1111/nmo.14948 (PMC11650404; doi:10.1111/nmo.14948)
Supplement: Supplementary file 1 — Appendix S1. [file NMO-37-e14948-s001.pdf]

Supplementary Material

Table 1S. *Rome III FC questionnaire symptoms before and after BF. All symptoms' frequency decreased after BF.*

	Before BF	After BF: No	After BF: Yes	Tot	p-value*
Straining during at least 25% of defecations	**No**	3	3	6	<0,001
	**Yes**	80	45	125	
	**Tot**	83	48	131	
Lumpy or hard stools in at least 25% of defecations	**No**	30	14	44	<0,001
	**Yes**	64	23	87	
	**Tot**	94	37	131	
Sensation of incomplete evacuation for at least 25% of defecations	**No**	0	1	1	<0,001
	**Yes**	85	45	130	
	**Tot**	85	46	131	
Sensation of anorectal obstruction/ blockage for at least 25% of defecations	**No**	44	6	50	<0,001
	**Yes**	47	34	81	
	**Tot**	91	40	131	
Manual maneuvers to facilitate at least 25% of defecations	**No**	70	3	73	0.021
	**Yes**	13	45	58	
	**Tot**	83	48	131	
Fewer than 3 defecations per week	**No**	100	0	100	<0,001
	**Yes**	21	10	31	
	**Tot**	121	10	131	

*McNemar Test;
Abbreviations: BF=Biofeedback; FC=Functional Constipation

Table 2S. *Association between manometric and BET outcomes and BF response. Patients who responded to BF significantly improves in Rome III FDD diagnoses and in BET.*

	Response to BF Yes	Response to BF No	p-value*
**Improvement after BF (DD pre → No DD post)**	48	7	<0.001
**No Improvement after BF (DD pre → DD post)**	0	13	
**Improvement after BF (IRP pre → No IDP post)**	29	2	<0.001
**No Improvement after BF (IRP pre → IDP post)**	0	4	
**Improvement after BF (BET failed pre→ BET succeeded post)**	82	0	<0.001
**No Improvement after BF (BET failed pre→ BET failed post)**	2	47	

*Chi-Square Test

Abbreviations: BF=Biofeedback; DD=Dyssynergic defecation; IDP=Inadequate defecatory propulsion, BET=Balloon expulsion test

Table 3S. *Rome III FC symptoms criteria frequency before and after BF therapy. In the table is reported the cumulative percentages of patients meeting the Rome III criteria for FC. After BF, 62.6% of patients showed 0 or only 1 criteria.*

	Before BF Therapy			After BF Therapy		
	**Freq.**	**Perc. (%)**	**Cumulative perc. (%)**	**Freq.**	**Perc. %)**	**Cumulative perc. (%)**
**0 criteria met**	0	0	0	63	48.1	48.1
**1 criteria met**	0	0	0	19	14.5	62.6
**2 criteria met**	6	4.6	4.6	1	0.8	63.4
**3 criteria met**	46	35.1	39.7	12	9.2	72.5
**4 criteria met**	43	32.8	72.5	13	9.9	82.4
**5 criteria met**	26	19.8	92.4	18	13.7	96.2
**6 criteria met**	10	7.6	100.0	5	3.8	100.0
**Tot**	131	100.0		131	100.0	

Abbreviations: BF=Biofeedback; Freq.=Frequency; Perc.=Percentage

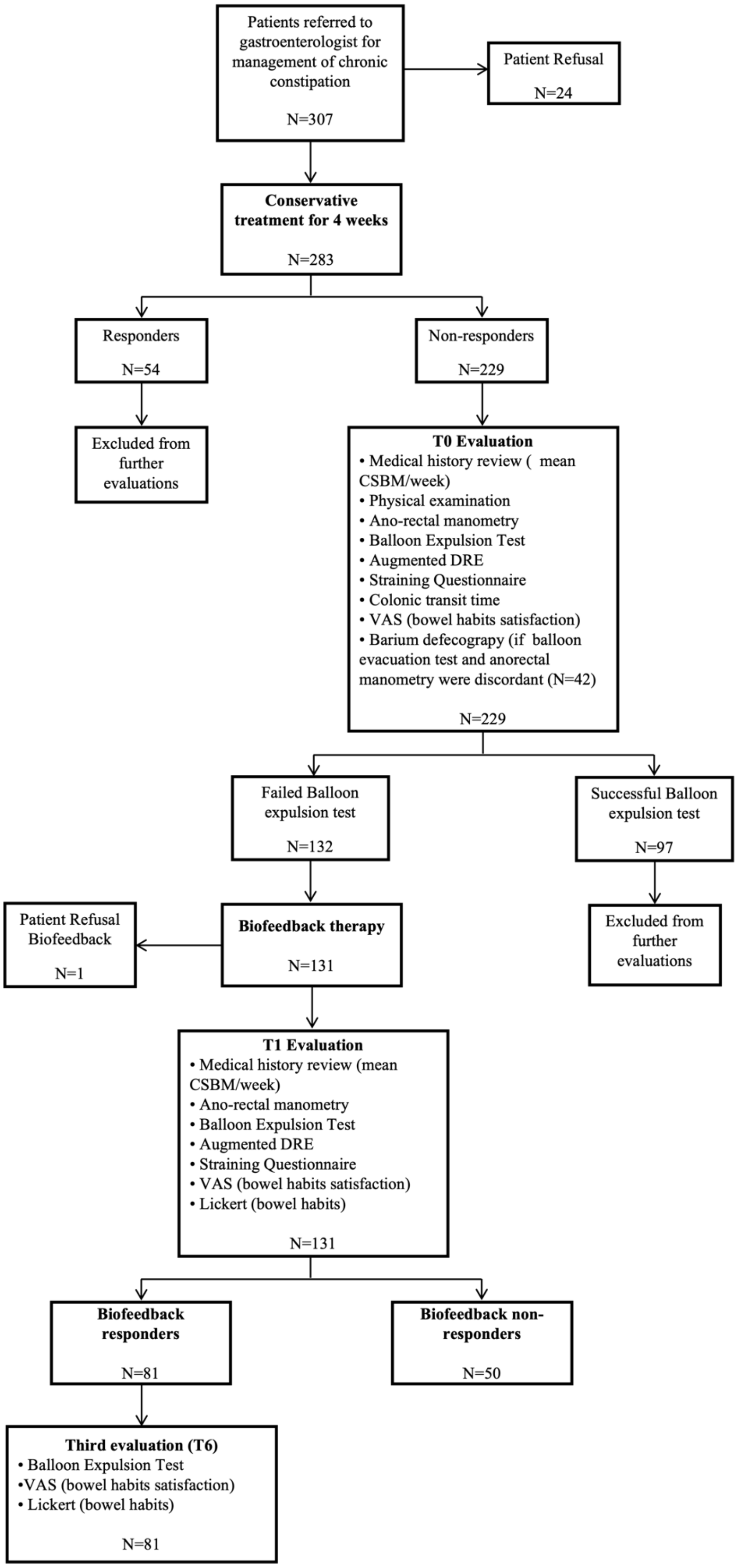

Figure 1S. Study Design

*Abbreviations: CSBM=Complete Spontaneous Bowel Movement; VAS=Visual Analogue Scale; Augmented-DRE=*digital rectal examination augmented by abdominal palpation during straining
